# RNA-binding proteins in tumor progression

**DOI:** 10.1186/s13045-020-00927-w

**Published:** 2020-07-11

**Authors:** Hai Qin, Haiwei Ni, Yichen Liu, Yaqin Yuan, Tao Xi, Xiaoman Li, Lufeng Zheng

**Affiliations:** 1 School of Life Science and Technology, Jiangsu Key Laboratory of Carcinogenesis and Intervention, China Pharmaceutical University, 639 Longmian Road, Nanjing, 211198 People’s Republic of China; 2 Guizhou Medical Device Testing Center, Guiyang, 550004 Guizhou People’s Republic of China; 3 Jiangsu Key Laboratory for Pharmacology and Safety Evaluation of Chinese Materia Medica, School of Pharmacy, Nanjing University of Chinese Medicine, Nanjing, 210023 People’s Republic of China

**Keywords:** RNA-binding proteins, RNA splicing, Polyadenylation, mRNA stability, mRNA localization carcinoma

## Abstract

RNA-binding protein (RBP) has a highly dynamic spatiotemporal regulation process and important biological functions. They are critical to maintain the transcriptome through post-transcriptionally controlling the processing and transportation of RNA, including regulating RNA splicing, polyadenylation, mRNA stability, mRNA localization, and translation. Alteration of each process will affect the RNA life cycle, produce abnormal protein phenotypes, and thus lead to the occurrence and development of tumors. Here, we summarize RBPs involved in tumor progression and the underlying molecular mechanisms whereby they are regulated and exert their effects. This analysis is an important step towards the comprehensive characterization of post-transcriptional gene regulation involved in tumor progression.

## Introduction

It is found that there are approximately 1914 human RNA-binding proteins (RBPs), accounting for 7.5% of protein-coding genes [1–3]. RBPs are highly species-conservative and play a key role in maintaining homeostasis of gene expression [4, 5]. Mounting evidences have shown that RBPs are involved in various important cellular processes, for instance, cell transport, localization, development, differentiation, and metabolism. Additionally, RBPs engage in almost every step of post-transcriptional regulation, supervise the formation and function of transcripts, and maintain cell homeostasis. Mechanistically, RBPs regulate RNA splicing, polyadenylation, mRNA stability, mRNA localization, and translation through interacting with coding and non-coding RNAs (ncRNAs) and other proteins [6, 7]. Increasing studies have shown that RBP-mediated RNA modifications are critical for cancer progression [8]. Furthermore, RBPs are abnormally expressed in different types of cancer and regulate the expression and function of oncogenes and tumor suppressor genes [9]. Therefore, it will provide new ideas or methods for finding novel targets of cancer treatment by revealing the mechanisms underlying RBP expression and the interaction between RBPs and their target RNAs.

### Structure of RBPs

**Fig. 1:**
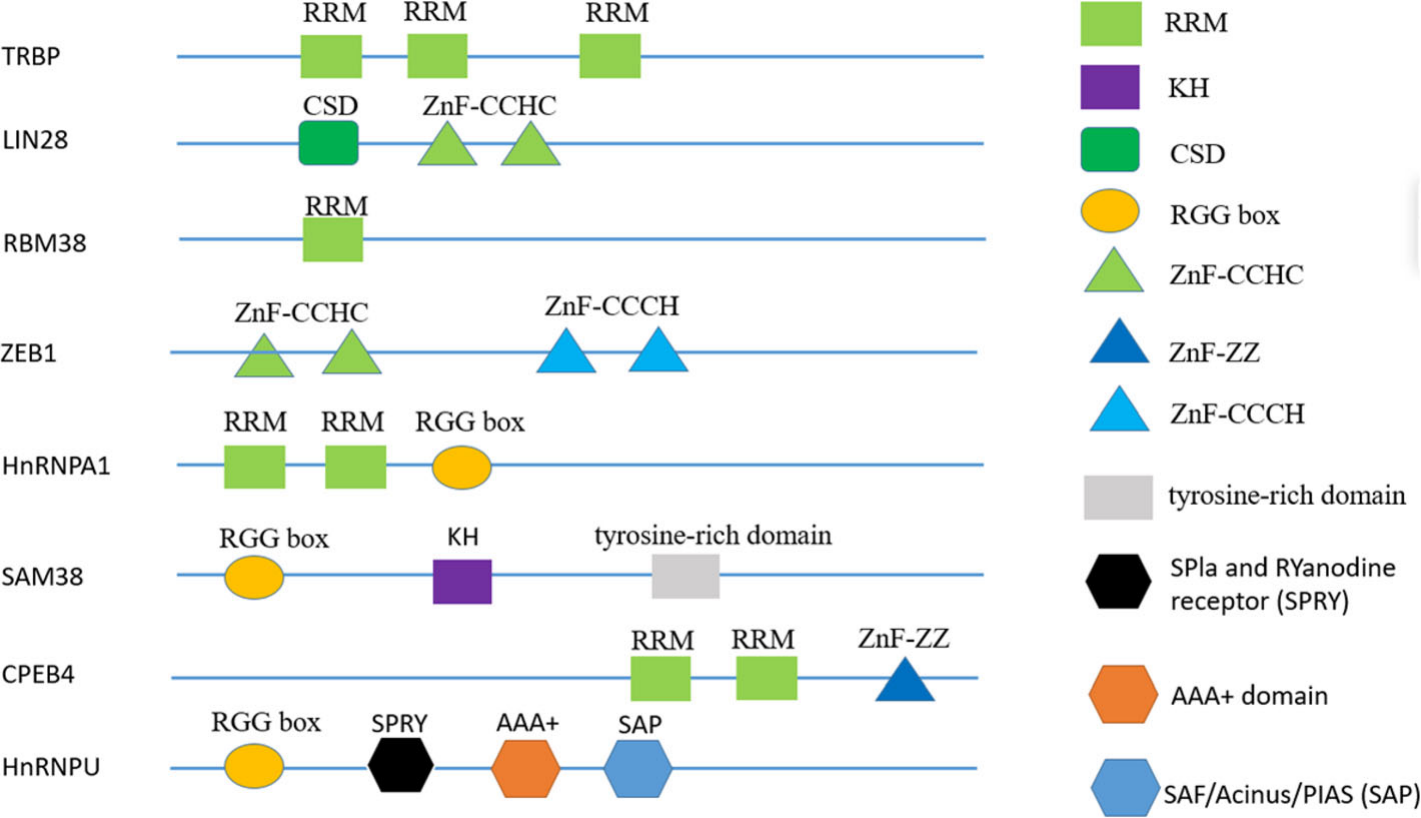
Currently, more than 50 domains of RBPs have been discovered. Here, we select the common RBP domains. Different domains are represented by colored boxes: RNA recognition motif (RRM), K homology (KH) domain, tyrosine-rich domain, arginine-glycine-glycine (RGG) motif, cold-shock domain (CSD), zinc fingers of the CCCH, CCHC, ZZ type etc

**Fig. 2:**
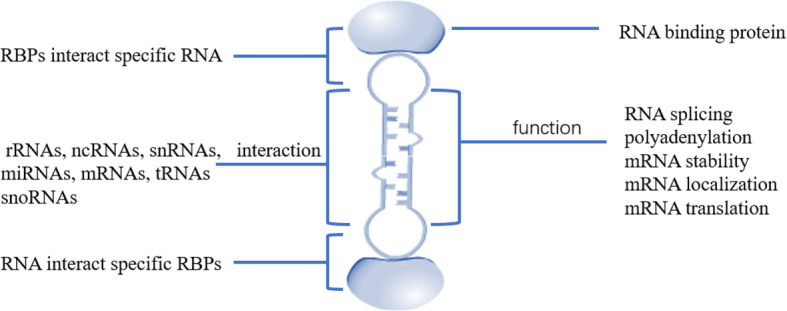
RBPs can interact with rRNAs, ncRNAs, snRNAs, miRNAs, mRNAs, tRNAs, and snoRNAs by binding to specific RNA-binding domains to perform specific biological functions

Many RBPs consist of multiple repetitive sequences that contain only a few specific basic domains. Structurally, common RNA-binding domains mainly include RNA-recognition motif (RRM), K homology (KH) domain, double-stranded RBD (dsRBD), cold-shock domain (CSD), arginine-glycine-glycine (RGG) motif, tyrosine-rich domain, and zinc fingers (ZnF) of the CCHC, CCCH, ZZ type etc. [10]. According to the different functions of RBPs in cells, RBPs can be divided into epithelial splicing regulatory proteins (ESRP1), cytoplasmic polyadenylation element binding protein family (CPEB1/2), Hu-antigen R (HuR), heterogeneous nuclear ribonucleoprotein family members (hnRNP A/D/H/K/M/E/L), insulin-like growth factor 2 mRNA family members (IMP1/2/3), zfh family of transcription factors (ZEB1/2), KH-type splicing regulatory protein (KHSRP), La ribonucleoprotein domain family members (LARP1/6/7), Lin-28 homolog proteins (Lin28), Musashi protein family (MSI1/2), Pumilio protein family (PUM1/2), Quaking (QK), RNA-binding motif protein family (4/10/38/47), Src-associated substrate during mitosis of 68 kDa (SAM68), serine and arginine rich splicing factor (SRSF1/3), T cell intracellular antigens (TIA1/TIAR), and Upstream of N-Ras (UNR) [10]. Figure 1 summarizes the basic domains of RNA-binding proteins (TRBP, LIN28, RBM38, ZEB1, HnRNPA1, SAM68, CPEB4) in certain tumors. In order to realize the diversity of RBP functions, these repetitive sequences can be arranged in different combinations for a specific RNA, and precise recognition of proteins can be achieved by rearranging these basic domains, which confers the diversity of RBP functions. Each basic domain recognizes RNA, but the domains of most of these proteins required multiple copies to comply with their functions (Fig. 2) [11]. This unique structure makes the binding specificity and affinity of RBPs be extremely improved. However, almost half of the RBPs have no specific binding sequences and structural elements. To explain this “non-specific” phenomenon, Jankowsky et al. [12] establish a model that integrates various parameters relating to the binding ability of RBPs, for example, the concentration ratio of RBPs to RNA in cells, the affinity distribution coefficient of RBPs, the constants of RNA substrate binding and dissociation rates, and the synergistic effect of RBPs and cofactors. Based on the above characteristics, RBPs can form a huge molecular interaction network and have a considerable impact on cell functions. Therefore, a systematic and functional study of RBPs will help us discover its role in tumors [13].

**Fig. 3:**
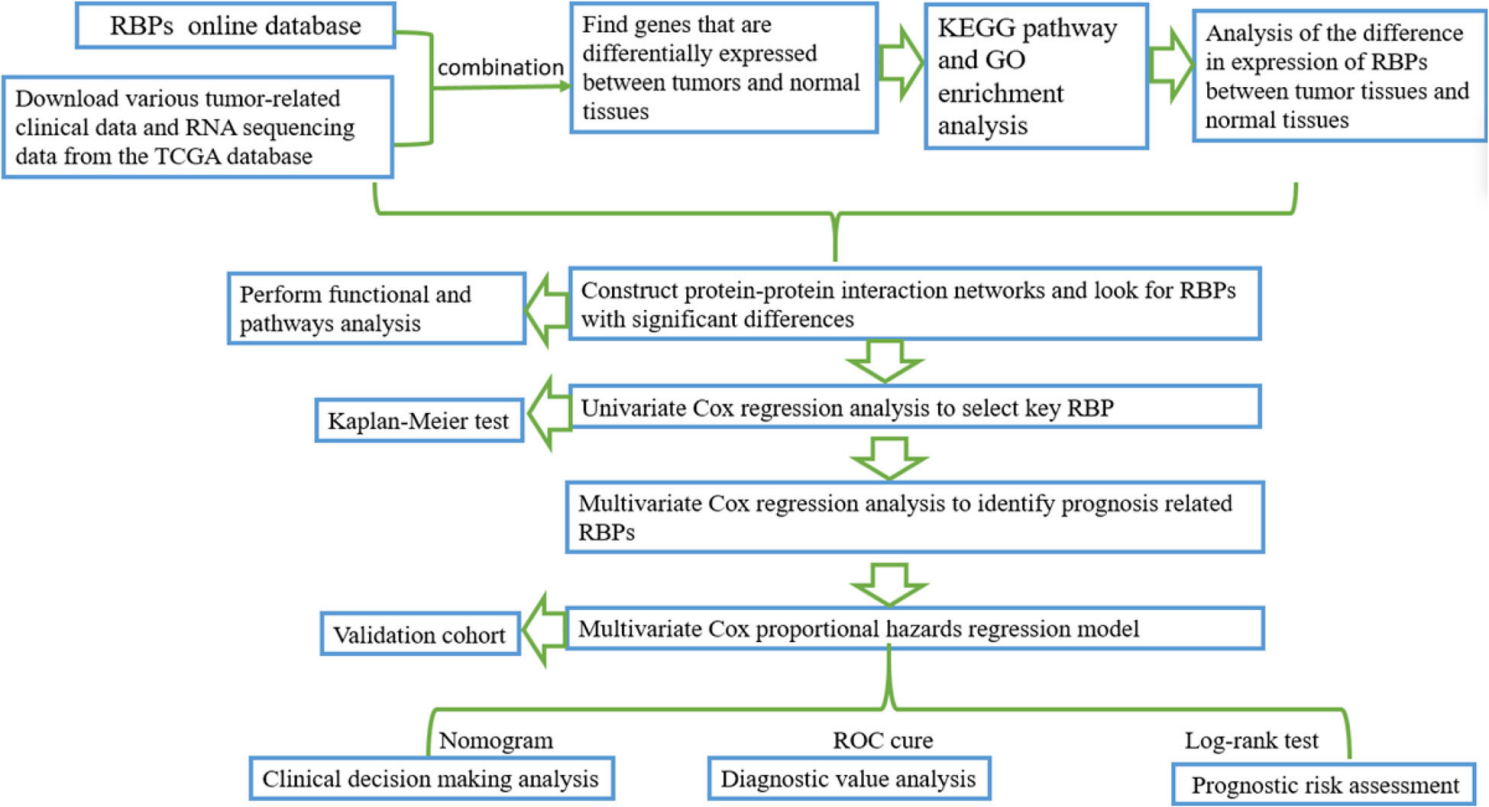
The whole process of RBP analysis

At present, the research methods of RBPs mainly include homopolymer binding method, ultraviolet cross-linking, SELEX, EMSA, whole-genome in vivo immunoprecipitation, and protein affinity purification [14]. In addition, there is an online database (RBPDB: http://rbpdb.ccbr.utoronto.ca/) including 1171 known RBPs, in which the users can browse by domain and species. And the Cancer Genome Atlas (TCGA) database can be used to download RNA high-throughput sequencing and clinical pathology data for determining the abnormal expression of RBPs between cancerous and normal samples [14]. Subsequently, the KEGG pathway and GO enrichment analysis of abnormal expression of RBPs can be used to systematically explore their potential functions and molecular mechanisms (Fig. 3) [15]. The discovered RBPs will promote our understanding of the molecular mechanism underlying tumor progression and provide potential biomarkers for clinical diagnosis and prognosis [16].

### Abnormality of RBPs in cancer

**Fig. 4:**
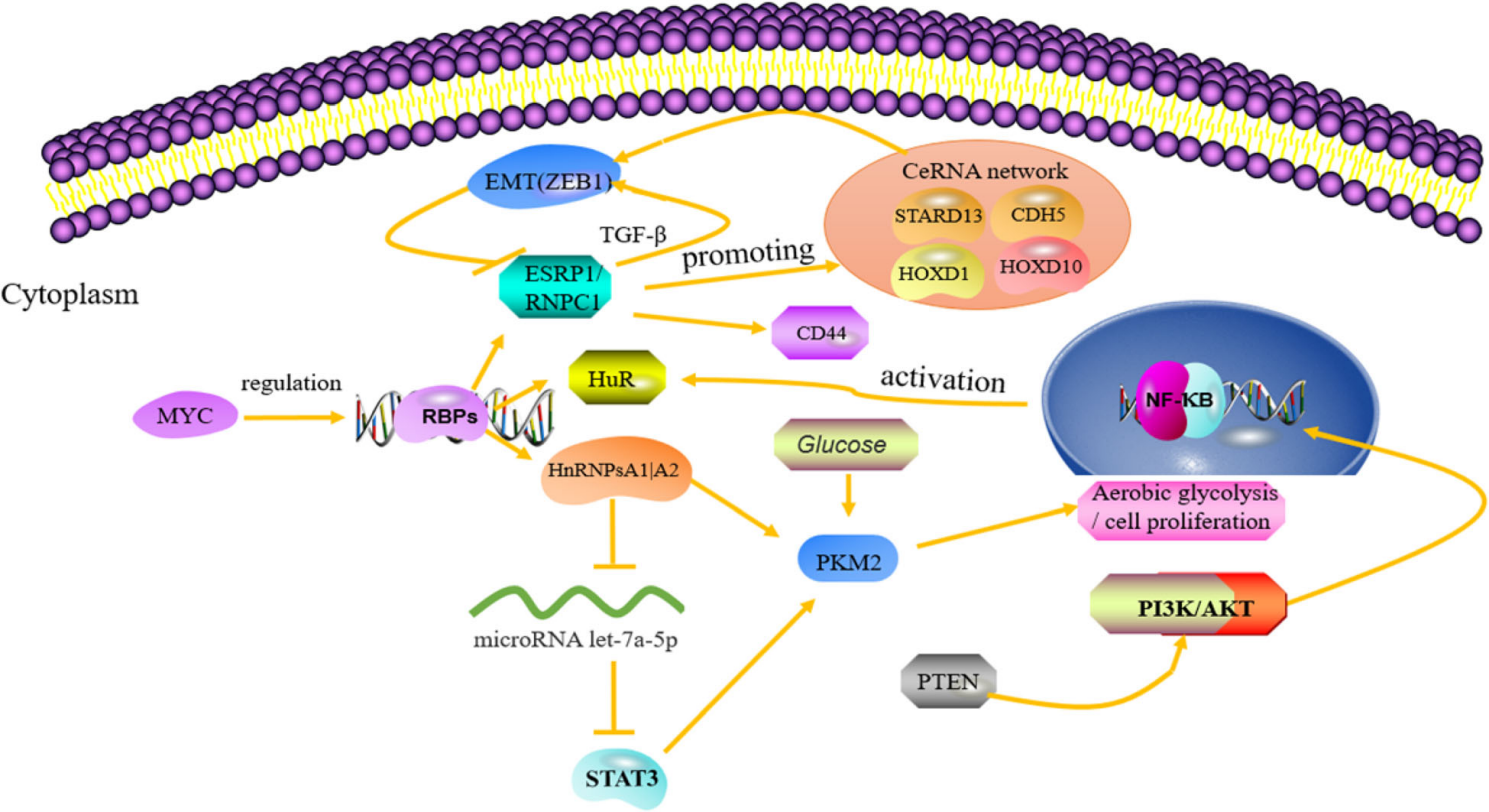
Signal pathways and metabolic pathways involved in abnormal RBP. HnRNP A1 /A2 is involved in the synthesis of M-type pyruvate kinase (PKM2), thereby enhancing the Warburg effect and let-7a-5p/Stat3/hnRNP-A1/PKM2 forms a cyclically regulated aerobic glycolysis. HuR regulates the PI3K/AKT/NF-κB signaling pathway. RNPC1 promotes the STARD13-mediated ceRNA network, thereby inhibiting the occurrence of EMT. The transcription factor ZEB1 protein can inhibit the mRNA level of epithelial splicing regulator protein 1 (ESRP1), resulting in the upregulation of the alternative spliceosome in the cell surface antigen CD44

Tumor is a complex heterogeneous disease and its pathogenesis is difficult to determine. Tumor is always accompanied with genetic mutations, which disturb the homeostasis of carcinogenic or tumor suppressor signaling pathways [10]. Perspectively, RBP holds structural and functional diversity essential for regulating several necessary cellular processes, for example, RNA splicing, modification, transport, localization, stability, degradation, and translation [17]. Some RBPs are commonly expressed and conservatively evolved to maintain the basic functions of cells. RBP disorders can cause various diseases, including cancer (Table 1) [55]. The target RNAs of RBP is very diverse. In addition to binding to exons, introns, and untranslated regions (UTR) of mRNA, RBP can also bind to non-coding RNA, such as microRNA (miRNA), transfer RNA (tRNA), siRNA, telomerase RNA, small nucleolar RNA (snoRNA), and splicing small nucleolar RNA (snRNA). Non-coding RNA forms secondary structures that can bind to RBPs and regulate multiple processes such as splicing, RNA modification, protein localization and secretion, and chromosome remodeling [56]. For example, the expression of long non-coding RNA (lncRNA) NEAT1 is upregulated in human CRC (colorectal cancer) tissues and related to the poor prognosis of CRC patients. NEAT1 interacts with RBP DDX5 and enhances its stability, subsequently activates the Wnt/β-catenin signaling pathway and promotes tumorigenesis [57]; the RBP TRBP is a molecular chaperone required for DICER1 to function. The mutation of TRBP results in abnormal expression of miRNA, and cancer cell proliferation and differentiation [58, 59]; HnRNPL is also a multifunctional RBP that interacts with long non-coding RNA (lncRNA) in different cancers. HnRNPL is mainly located in the nucleus, but it can also exert effects in the cytoplasm via post-transcriptionally regulating lncRNA expression [60, 61]. Additionally, the oncogenic transcription factor MYC can upregulate the mRNA levels of heteronuclear ribonucleoproteins (hnRNP) A1 and A2 in a variety of malignant tumors, and hnRNPs facilitate the synthesis of M-type pyruvate kinase (PKM2), thus strengthening the Warburg effect [24, 25, 62–64]. In gastric cancer cells, the PI3K/AKT/NF-κB and METTL3/ZMYM1/E-cadherin signaling pathways can promote the transcription of RBP human antigen R (HuR), which is engaged in increased cell growth and anti-apoptotic capability [28, 30, 65]. Furthermore, epithelial-mesenchymal transition (EMT)—specific transcription factor ZEB1 protein can directly inhibit the mRNA level of epithelial splicing regulatory protein 1 (ESRP1), which leads to the increased expression of various spliceosomes of cell surface antigen CD44 and induces lung, breast, and pancreatic cancer stem-like and aggressive cells [35–37]. In breast cancer, RNA-binding motif protein 38 (RBM38) participates in the TGF-β-induced EMT pathway and stabilizes the tumor suppressor gene PTEN transcript to enhance expression [40–42]. Moreover, our previous study indicates that the RBM38 can suppress breast cancer metastasis through facilitating STARD13-related competing endogenous RNAs (ceRNA) network (Fig. 4) [66]. In hepatocellular carcinoma (HCC), dysregulation of RBPs disrupts the transcriptome balance within tumor cells, thereby driving tumorigenicity. For example, NELFE and PTBP3, as a kind of RBP, promote the malignant growth and metastasis of HCC cells by regulating the binding of MYC and splice variants (NEAT1_1, NEAT1_2, and miR-612) to the target promoter and the stability of MYC-regulated mRNAs [46, 50]. In summary, the abnormal function of RBPs has a significant impact on tumor phenotypes.

**Table 1:** Role of RBPs in tumors

RBP	The basic mechanism of RBPs regulation	Tumor type	Established target	Up- or downregulation	Model or cells	Mechanism/signaling pathway/conclusion	Biological functions	Refs
TRBP	mRNA translation, mRNA stability	Breast carcinomas, colorectal cancer, endometrial cancer	Amyloid precursor protein (APP), ZNF395	Up- or downregulation	Human colorectal and endometrial cancer cell lines	PKR pathway	Promotes or inhibits cell proliferation and invasion	[18–20]
hnRNP E1	mRNA transcription, mRNA stability, mRNA transport, alternative RNA splicing	Human hepatoma cell, melanoma, breast cancer	PNUTS, miR-205	Downregulation	Clinical specimens of human hepatocellular carcinoma (HCC) A549 MDA-MB-231	The deletion of hnRNP E1 in liver cancer contributes to the formation of metastatic phenotype. hnRNPE1 impedes the shearing of lncRNA-PNUTS, thereby inhibiting tumor cell migration, invasion, and metastasis.	Inhibits cell proliferation, invasion, and metastasis EMT	[21, 22]
hnRNPL	Alternative RNA splicing	Prostate cancer	CTBP1, ROR2, STX3	Upregulation	CRISPR Cas9-LNCaP, CWR22Rv1, DU145, and PC3 cells	HNRNPL regulates circular RNA formation in human prostate cancer.	Promotes cell proliferation, invasion, and metastasis	[23]
hnRNP A1/A2	Alternative RNA splicing	Glioma, breast cancer, hepatocellular carcinoma	PK-M1/M2, SIRT1, SIRT6, let-7a, c-Myc, Stat3	Upregulation	Glioma xenograft model, breast cancer clinical samples, HepG2 cells stably expressing hnRNP A1 or 4KR	Let-7a/c-Myc/hnRNPA1/PKM2 signaling. Let-7a-5p/Stat3/hnRNP-A1/PKM2 signaling pathway. Sirtuin-mediated deacetylation of hnRNP A1 inhibits HCC cell proliferation and tumorigenesis in a PKM2-dependent manner.	Promotes apoptosis, proliferation, migration, and invasion	[24–26]
hnRNPK	mRNA transcription, mRNA translation	Non-small-cell lung cancer	MAP 1B-LC1	Upregulation	NSCLC clinical samples, adjacent non-tumor tissues	Interaction of hnRNP K with MAP 1B-LC1 promotes TGF-β-mediated EMT in lung cancer cells.	Promotes proliferation, EMT	[27]
hnRNPC	Alternative RNA splicing	Breast cancer, gastric cancer	RIG-I, 5B2	Upregulation	Constructed CRISPR/Cas9-hnRNPC MCF7 and T47D cell lines; gastric cancer cell lines resistant to 5-fluorouracil (5FU), paclitaxel (TA), and cisplatin (DDP)	Inhibition of HNRNPC prevented the proliferation and tumorigenesis of MCF7 and T47D and activated the type I interferon response. HNRNPC as a candidate biomarker for chemoresistance in gastric cancer	Promotes proliferation, tumorigenesis	[28, 29]
HuR	subcellular localization, mRNA stability, mRNA translation	Gastric cancer, breast cancer, colon cancer, lung cancer, varian cancer	CCNA1/B1/E1, MDM2, MYC, PTMA, SIRT1, SNAIL, VEGF	Upregulation	Clinical samples of various tumor tissues MDA-MB-231, MCF-7, H1299, A549, MRC-9, CCD16	HuR is usually activated through the PI3K/AKT/NF-kB pathway. Circ-HuR serves as a tumor suppressor to inhibit CNBP-facilitated HuR expression and gastric cancer progression. MiR-155-5p controls the migration of colon cancer cells through HuR post-transcriptional regulation. Integrin β1/FAK/ERK signaling	Exerts proliferation anti-apoptotic effects	[30–34]
ZEB1	mRNA stability	Multiple tumors	CCR2, CCL2, miR-200, miR-203, MMPs, CDH1, IL6IL8, PDL1, INK4A/B, MSRB3	Upregulation	Mouse models and human samples	ZEB1 has a pleiotropic effect in cancer, promoting the dynamic process of reversible transformation of tumor cells between metastable states.	Foster EMT, stemness, invasiveness	[35–39]
RBM38	mRNA stability, mRNA translation, post-transcriptional regulation, mRNA splicing	Colorectal cancer, acute myeloid leukemia, renal cell carcinoma, hepatocellular carcinoma	PTEN, ZO-1, STARD13, CDH5, HOXD10, HOXD1, CDKN1A, LATS2 P53, Mdm2	Downregulation	Cell lines and clinical samples of various tumors	The potential tumor suppressor gene RBM38 has been identified in various tumors.	Inhibit EMT, stemness, invasiveness	[40–45]
PTBP3	Alternative RNA splicing, mRNA stability, RNA transport, RNA translation, RNA decay	Hepatocellular carcinoma, breast cancer, gastric cancer	NEAT1, pre-miR-612, ZEB1, CAV1	Upregulation	Human HCC tissues, gastric cancer cells (MKN45 and SGC7901) Clinical samples MCF-7, MDA-MB-453/231	PTBP3 regulates the balance of splicing variants (NEAT1_1, NEAT1_2, and miR-612) in HCC. PTBP3 as a regulator of EMT that acts by governing expression of ZEB1. PTBP3 may regulate CAV1 through alternative splicing and become a metastasis gene for gastric cancer.	Promoted HCC cell proliferation and metastasis both in vitro and in vivo regulation EMT	[46–48]
PTBP1	RNA transport, RNA translation	Acute myeloid	FLT3	Upregulation	FLT3-ITD-positive cells, FLT3-ITD-negative cells	circMYBL2 regulates FLT3 translation by recruiting PTBP1 to promote FLT3-ITD AML progression.	Promotes proliferation and differentiation	[49]
NELFE	mRNA stability, RNA translation	Hepatocellular carcinoma	MYC-related genes, SYNGR2	Upregulation	Clinical HCC samples	NELFE is an oncogenic protein that causes imbalance in the HCC transcriptome by regulating MYC signaling.	Promoted HCC cell proliferation and metastasis	[50]
LIN28	mRNA transcription, mRNA translation	Multiple tumors	Let-7 family members, PD-L1	Upregulation	Clinical samples of various tumor tissues	Crosstalk between LIN28A/LIN28B and let-7 loops and certain oncogenes (such as MYC, RAS, PI3K/AKT, NF-κB, and β-catenin) to regulate the characteristics of cancer. LIN28/let-7/PD-L1 pathway	Poor prognosis, increased cellular proliferation	[51, 52]
HNRNPU	Alternative RNA splicing, mRNA stability, mRNA metabolism mRNA transport	Neuroblastoma	HNF4A-AS1, CTCF	Upregulation	MCF 10A, HEK293T, NB cell lines	HNF4A-AS1/hnRNPU/CTCF axis	Promote aerobic glycolysis and NB progress	[53, 54]

#### NcRNA-mediated RBP disorders

**Fig. 5:**
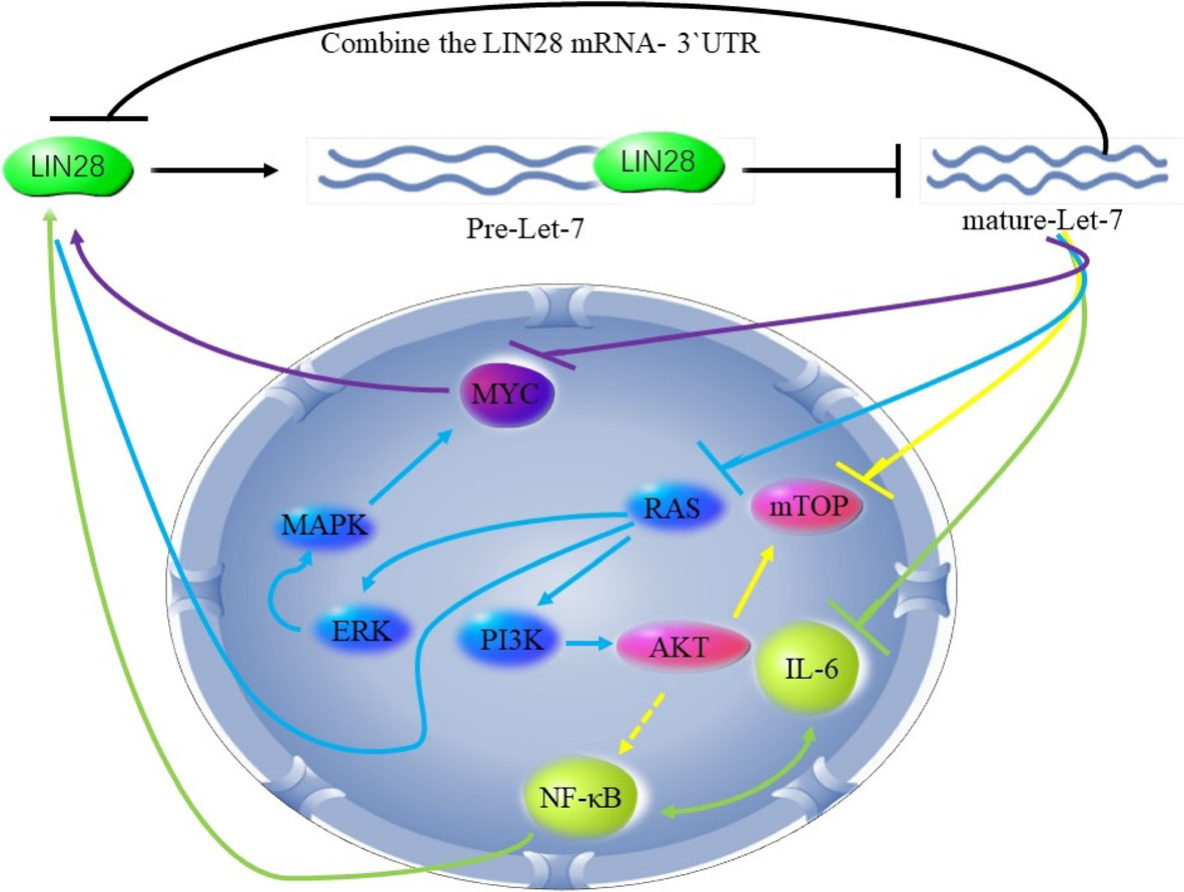
The LIN28/let-7 two-way negative feedback mechanism is involved in the processing of let-7 precursor miRNA into mature miRNA and combines with some cytokines (such as the SCR family, MYC family, and NF-κB) to form a complex factor regulatory system involved in cancer occurrence

NcRNA-mediated post-transcriptional regulation is closely related to the expression of RBPs. For example, LIN28 is an RBP mainly composing of two subunits, LIN 28A and LIN 28B, both of which have a similar structure and function. LIN28 is often dysregulated in tumor cells, and the upregulation of LIN28 is inversely related to the poor prognosis of advanced cancer. LIN28 inhibits the processing of let-7 precursor miRNA into mature miRNA in poorly differentiated cells, increases PD-L1 transcription and translation, and then promotes the growth and immune escape of various cancer cells [67]. Conversely, the let-7 family members can inhibit LIN28 expression [68]. Meanwhile, let-7 can bind to PD-L1 3′UTR, thereby inhibiting PD-L1 expression and enhancing the immune response of cells [51]. Therefore, there is a mutual negative feedback regulation between LIN28 and let-7 families. Furthermore, there are numerous evidences showing that this LIN28/let-7 two-way negative feedback can be combined with some cytokines (such as the SCR family, MYC family, and NF-κB) to form a complex regulatory system involving in tumor development (Fig. 5) [69–72]. In addition, RBP Musashi1 (MSI1) is restrained by a class of tumor suppressor miRNAs, including miR-34a, miR-101, miR-128, miR-137, and miR-138, and this inhibition reduces glioblastoma progression [73, 74]. What’s more, lncRNA is also engaged in the regulation of RBPs. For instance, NEAT1 is a lncRNA contributing to the formation of paraspeckle nuclear structures. Knockdown of NEAT1 results in the decreased expression of RBPs including hnRNP A2 and IGFBP3, both of which can inhibit tumor proliferation, migration, and invasion in liver cancer cells [75]. Moreover, lncRNA MT1JP enhances the p53 translation by combining with RBP TIAR, thereby regulating p53-related pathways, such as cell cycle, apoptosis, and proliferation [76]. In glioblastoma, LINC00470, a long non-coding RNA located on the chromosome, belongs to an oncogene RNA. LINC00470 directly binds to FUS and anchors FUS in the cytoplasm, activates FUS protein and forms a stable complex with AKT, activates AKT nuclear translocation and thereby promotes tumorigenesis [77].

#### Post-translational modification-mediated RBP disorders

**Fig. 6:**
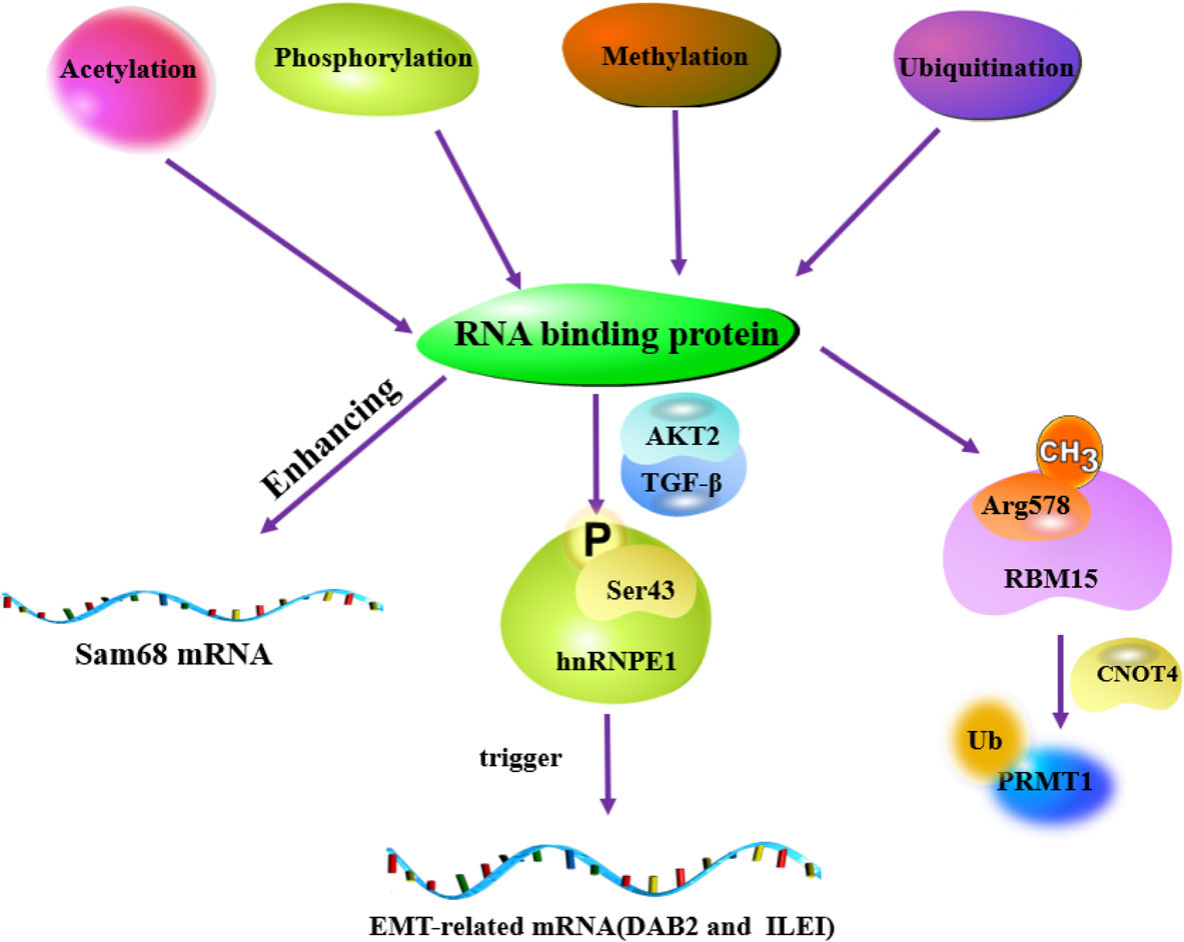
RBP post-translational modifications (PTMs) (acetylation, phosphorylation, methylation, and ubiquitination) are involved in important biological processes. Different PTMs regulate the abundance of RBP, subcellular localization, and different protein kinases and signal transduction pathways. Abnormal PTM promotes carcinogenesis, apoptosis, tumorigenesis and cancer progression by changing the binding ability of IGF2BPs to mRNA, and regulating the protein activity, stability, and localization of hnRNPE1, RBM15 and Sam68

The abnormal function of RBP is also caused by abnormal post-translational modifications (PTM) in tumors, originates from multiple signaling pathways and activates the spatial conformation of enzymes, thereby inducing changes in its functional chemical groups, including acetylation, phosphorylation, methylation, ubiquitination, and N6-methyladenosine methylation (m6A) (Fig. 6) [78]. PTM can change the binding ability of RBP and play a key role in regulating protein activity, stability, positioning, interaction, or folding [79, 80]. It is worth noting that the PTM of RNA-binding elements in RBP are particularly prominent. The abnormal modification of this site may be one of the main factors leading to RBP dysfunction in tumors [81]. For example, as a member of the TAR family, the acetylation level of RBP Sam68 is higher than that of normal cells, thereby enhancing the binding ability of Sam68 to target genes whose expression is closely related to tumor cell proliferation [82, 83]. In non-invasive breast epithelial cells, activation of the TGFβ/AKT2 pathway phosphorylates serine 43 of hnRNPE1, which triggers translation of EMT-related mRNAs (DAB2 and ILEI) and facilitates EMT, metastasis, and invasion [84–86]. GSK3 can mediate phosphorylation of RBM38 at serine 195, causing RBM38 conformation to change, which prevents it from binding to eukaryotic translation initiation factor 4E (eIF4E) on p53 mRNA in breast and colon cancer cells [87–89]. In acute megakaryoblastic leukemia, the protein arginine methyltransferase 1 (PRMT1) is highly expressed and can methylate R578 residue on RBM15 and promotes PRMT1 ubiquitination-mediated degradation by E3 ligase (CNOT4). Notably, RBM15 can regulate a series of genes related to megakaryocyte production [90, 91]. RBP’s PTMs are involved in important biological processes, including carcinogenesis, apoptosis, tumorigenesis and cancer progression, cell division and cell response to stress drugs. Different types of RBP have different effects on protein transport, secretion, function and elimination. RBP can dynamically change the regionalization, transport and physical interaction of key molecules that regulate different cellular processes. Therefore, PTM enables RBP to cause rapid changes in gene expression programs. For example, the affinity of RBP for target RNA or DNA and change the interaction of RBP with other proteins. Different PTMs regulate RBP abundance, subcellular localization, and multiple protein kinases and signaling pathways. Through the comprehensive integration of genetic, biochemical, molecular biology, and structural analysis of RBP protein-protein and protein-RNA complexes, we hope to find new targets for therapeutic intervention.

#### Transcriptional regulation-mediated RBP disorders

**Fig. 7:**
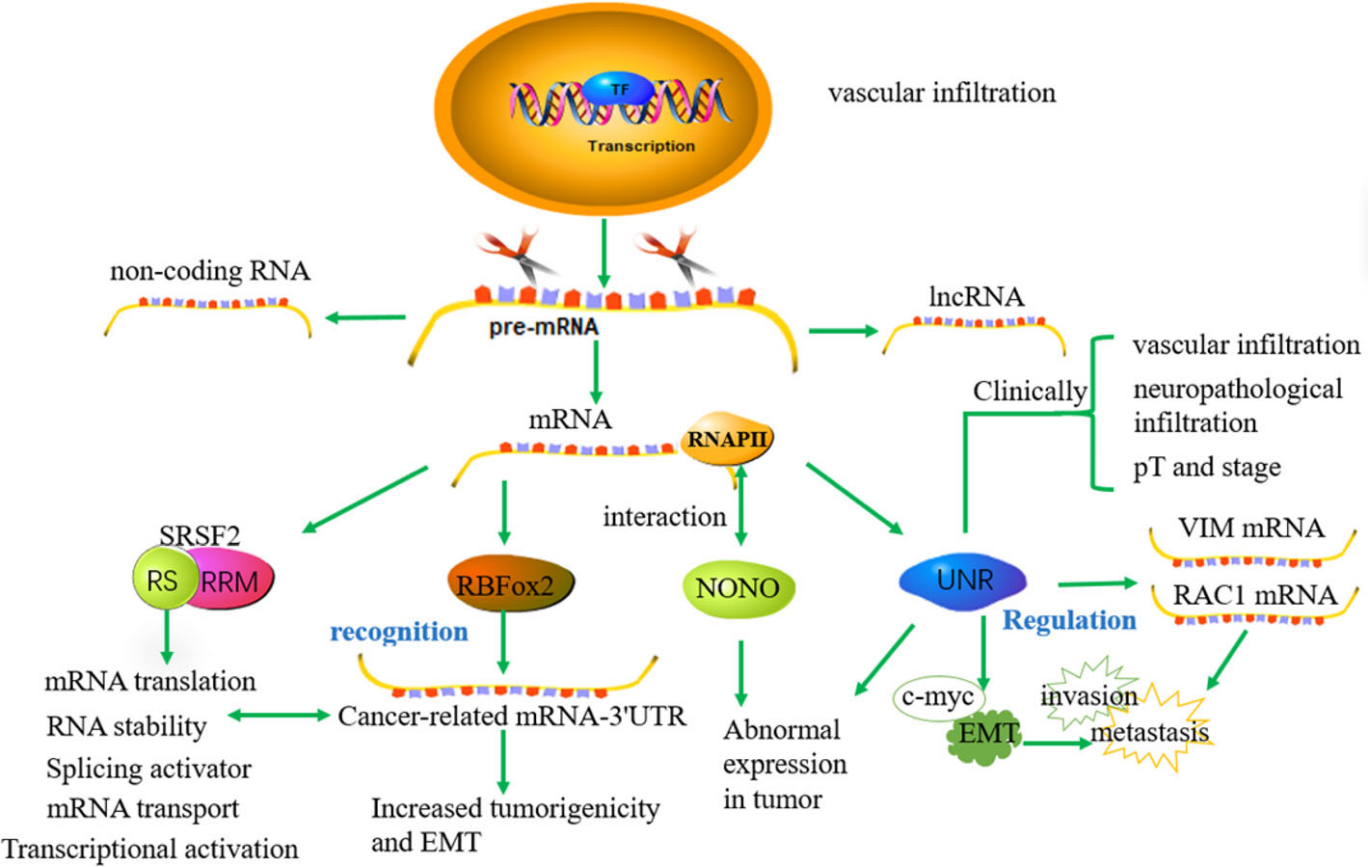
In addition to producing protein-encoding mRNA, genomic DNA also produces many non-coding RNAs, including long non-coding RNA (lncRNA) and miRNA, many of which are directly involved in transcription control. The occurrence of RNA transcription regulation involves multiple RNA-binding proteins (SRSF2, RBFox2, NONO). The abnormal function of typical splicing regulators in transcription is closely related to the pathogenesis of cancer

In addition to produce protein-coding mRNA, mammalian gene expression also produces many ncRNAs, including lncRNA, many of which are directly involved in transcription control [92]. The occurrence of RNA transcriptional regulation involves multiple RBPs participating in the recruitment of specific RNA-binding proteins (RBPs). The transcriptional regulation of RBPs is a fast and effective method that can continuously adapt transcription and translation of the proteome to the environment. Indeed, many RBPs have a direct role in transcription, and their ectopic regulation can lead to tumorigenesis. For example, the abnormal function of typical splicing regulators in transcription is closely linked to the pathogenesis of cancer. Importantly, these cancer-specific splicing variants are usually upregulated in tumors, which help tumor cell survival and cancer progression and also predict the survival of cancer patients (Fig. 7). Common specific splicing variants are SRSF2, RBFox2, NONO, etc. [93, 94]. SRSF2 belongs to SR protein and is composed of RRM domain and RS domain. Its main functions are splicing activator, transcriptional activation, RNA stability, mRNA transport and translation [95]. SRSF2 protein is highly expressed in hepatocellular carcinoma and predicts a poor prognosis of patients. SRSF2 can stimulate the expression of clinical HCC-related splice variant GCH1 at the transcription level [93]. GCH1 splice variants play a key role in cancer cell survival and tumorigenic potential [96]. Therefore, SRSF2 regulates the tumorigenesis of liver cancer cells by controlling the expression of cancer-associated splice variants. Stress particles (SGs) are stagnant translation initiation complexes that contain untranslated mRNA and RNA-binding protein (RBP). RBP fox-1 homolog 2 (Rbfox2) is part of SG and belongs to typical RBP [97]. It regulates variable pre-mRNA splicing in the nucleus and binds to retinoblastoma 1 (Rb1) mRNA [98]. Rbfox2 migrates to the cytoplasm when subjected to external pressure [98]. The RNA recognition motif domain of the Rbfox family protein binds to the 3′UTR region of the target mRNA related to cancer development, which contributes to the stability of the mRNA and thus promotes the progression of cancer [99, 100]. NONO is a versatile RBP that mainly plays a biological role in the nucleus [101]. In addition to the characteristics of nucleic acid binding, NONO can also interact with the terminal end region of the RNA polymerase II subunit to participate in pre-mRNA synthesis and treatment [102]. NONO expression is abnormal in prostate cancer, colorectal cancer, breast cancer and melanoma, which indicates that NONO may cause tumors [103–106]. In addition, at the transcription level, upstream-of-N-Ras (UNR) also plays an important role. UNR named CSDE1, belongs to a conservative RBP consisting of five cold-shock domains (CSDs). The specific domains of UNR can bind to mRNA (IRES) and thus change its structure to a more powerful translation [107]. UNR has been reported to regulate the expression of c-fos, c-Myc and other proto-oncogenes [108]. UNR is mainly located in the cytoplasm to regulate the transcription, translation and stability of mRNA [109]. In melanoma, UNR is usually highly expressed in melanoma and associated with invasion and metastasis by activating the extension of VIM and RAC1 mRNA [110]. Similarly, UNR is overexpressed in colorectal cancer (CRC) cells and patients, and closely related to cell survival, invasion, anti-apoptosis and poor prognosis by regulating EMT and c-MYC expression [108]. Clinically, the expression of UNR is related to the poor prognosis of resection PCDA patients. The expression of UNR is of great significance to clinicopathological features such as vascular infiltration, neuropathological infiltration, pT, and stage [111]. Based on the above research result, the UNR/CSDE1 axis might be as a prognostic biomarker for resection PCDA patients.

### The molecular mechanisms underlying RBP roles in cancer progression

There are various mechanisms contributing to RBP-mediated regulation on cancer progression, including alternative splicing, polyadenylation, stability, subcellular localization and translation.

#### Alternative splicing

**Fig. 8:**
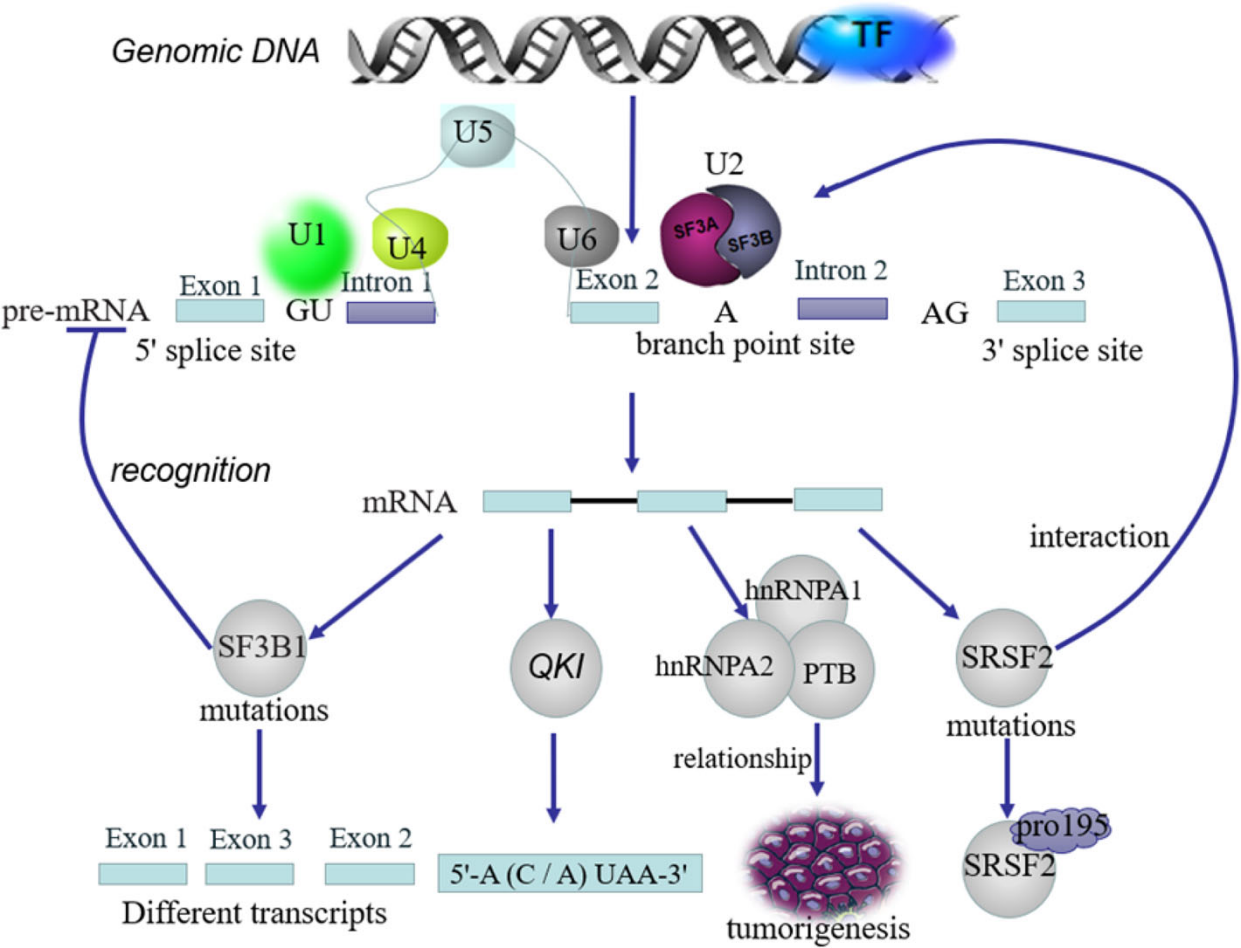
Alternative splicing contributes to protein diversity and mRNA stability. Abnormal or erroneous splicing of RBPs is one of the causes of cancer. Some RBPs can form complexes with core proteins and combine with splicing, increase or decrease the activity of spliceosomes and jointly control the splicing of tumor cell molecules. SF3B1, hnRNP, and SR proteins are selective splicing regulators. They recognize spliceosomes by binding to spliceosomes, which in turn regulates splicing. Abnormalities in alternative splicing regulators are often associated with tumorigenesis

Alternative splicing is a major post-transcriptional regulatory mechanism contributing to protein diversity and mRNA stability. The main types of aberrant splicing in tumors are constitutive splicing, exon skipped or included, alternative 5_ splice-sites, alternative 3_ splice-sites, intron retention and mutually exclusive exon [112]. Abnormal or erroneous shearing is one of the principal causes of the abnormal function of RBPs and can promote cancer occurrence [113–116]. Some RBPs can form complexes with core proteins linked to splicing and together control the splicing of molecules in tumor cells. There are also some RBPs whose binding sites are tied to the regulation of exon. These proteins can increase or decrease the activity of spliceosome in various situations (Fig. 8) [103, 117, 118]. For example, SF3B1, hnRNP and serine/arginine (SR)-rich selective splicing regulators that recognize spliceosomes by binding to spliceosome point, which in turn regulates splicing [93, 119–122]. Additionally, SF3B1 missense mutations are not able to recognize pe-mRNA, leading to differential splicing of transcripts [123–125]. The most frequently mutated (SR) protein is SRSF2, whose mutation site is proline 195, which holds the RNA-binding specificity [121, 126–128]. The anomalous expression of hnRNPs and SRs in numerous cancers suggests that the splicing function of these two proteins plays a significant role in tumor progression. Additionally, RBP QKI in STAR family members is a splicing factor. Compared with normal tissues, the expression of QKI is significantly reduced in lung cancer, which is associated with a poor prognosis [129–132]. QKI binds to QKI response element [QRE, 5′-A (C/A) UAA-3] and exerts multifunctional effects on target RNAs, including localization, stability, translation efficiency, and microRNA (miRNA) processing [133, 134]. In normal cells, QKI can compete with the core splicing factor SF1, selectively suppress the splicing of exon 12 of NUMB mRNA, and upregulate the expression of NUMB isoforms, thereby inhibiting cell proliferation and preventing the activation of Notch signaling pathway [135, 136]. RBM10 is another splicing factor. Like QKI, RBM10 expression is significantly reduced in lung cancer cells. By inhibiting the splicing of exon 9 of NUMB mRNA, RBM10 increases the expression of NUMB. RBM10 can also suppress cell proliferation though regulating the Notch signaling pathway [137–139]. HnRNPA1/2 and PTB splicing factors hold the opposite functions and their inhibition of splicing is related to tumorigenesis [62]. The expression of hnRNPA1/2 is relatively high in gliomas and associated with a poor prognosis [140]. By binding to the promoter of hnRNPA1/2 and PTB, c-Myc upregulates PKM to promote tumor cell proliferation and transformation [24, 62, 140, 141]. After hnRNPA2 expression is upregulated and the exon 7 of CFLAR mRNA, exon 12a of BIN1 mRNA and exons 6-8 of WWOX mRNA cannot be spliced, resulting in the conversion of these tumor suppressor proteins into anti-apoptotic protein isomers in human astrocytoma cells [140]. Similarly, RBP hnRNA1 can inhibit the splicing of exon 11 of RON mRNA, which promotes this conversion of the tyrosine kinase receptor into oncoproteins and controls the occurrence of tumor invasion and migration [140]. In human tumors, defects of alternative splicing often occur, which may be due to mutations in splicing regulatory elements of specific cancer-related genes, or changes in the process of splicing regulation. Alternative splicing can also produce a new class of tumor proteins or tumor suppressors, which promotes disease progression by regulating RNA subtypes associated with tumor signaling pathways. Dysregulation of alternative splicing is a basic process of cancer. Understanding the regulatory factors of the splicing mechanism is a key step in understanding the role of alternative splicing in cancer, and provides new targets and biomarkers for the treatment of tumors.

#### Alternative polyadenylation (APA)

In most eukaryotes, RBPs attend an important part of pre-mRNA post-processing, adding a poly (A) tails at the 3′ end of the mRNA. This process corresponds to the addition of a long string of adenylate on the 3′ end of the transcript. The pre-mRNA 3′ end processed complexes are mainly composed of CPSF, CSTF, CFI, CFII, CTD and RNAPII. About 85 proteins engage in this process [142, 143]. This process is necessary for mRNA maturation, stability, nuclear transport and efficient translation [144, 145]. The length of the poly (A) tails must be an extremely important biological characteristic and is highly conservative in evolution in diverse species and genes. Similar to splicing, most selective APA is located in the intron region upstream of the last exon and its role is to generate non-coding transcripts or transcripts with truncated coding regions. APA makes multiple different transcripts, and the coding region and 3′untranslated region (3′UTRs) of these transcripts are different. By amending the coding sequence, APA affects the function of the protein. For 3′UTRs, APA can alter its length, thereby regulating the stability, subcellular position and translation efficiency of target mRNAs. In cancer, abnormality at the 3′ end of pre-mRNA often occurs.

**Fig. 9:**
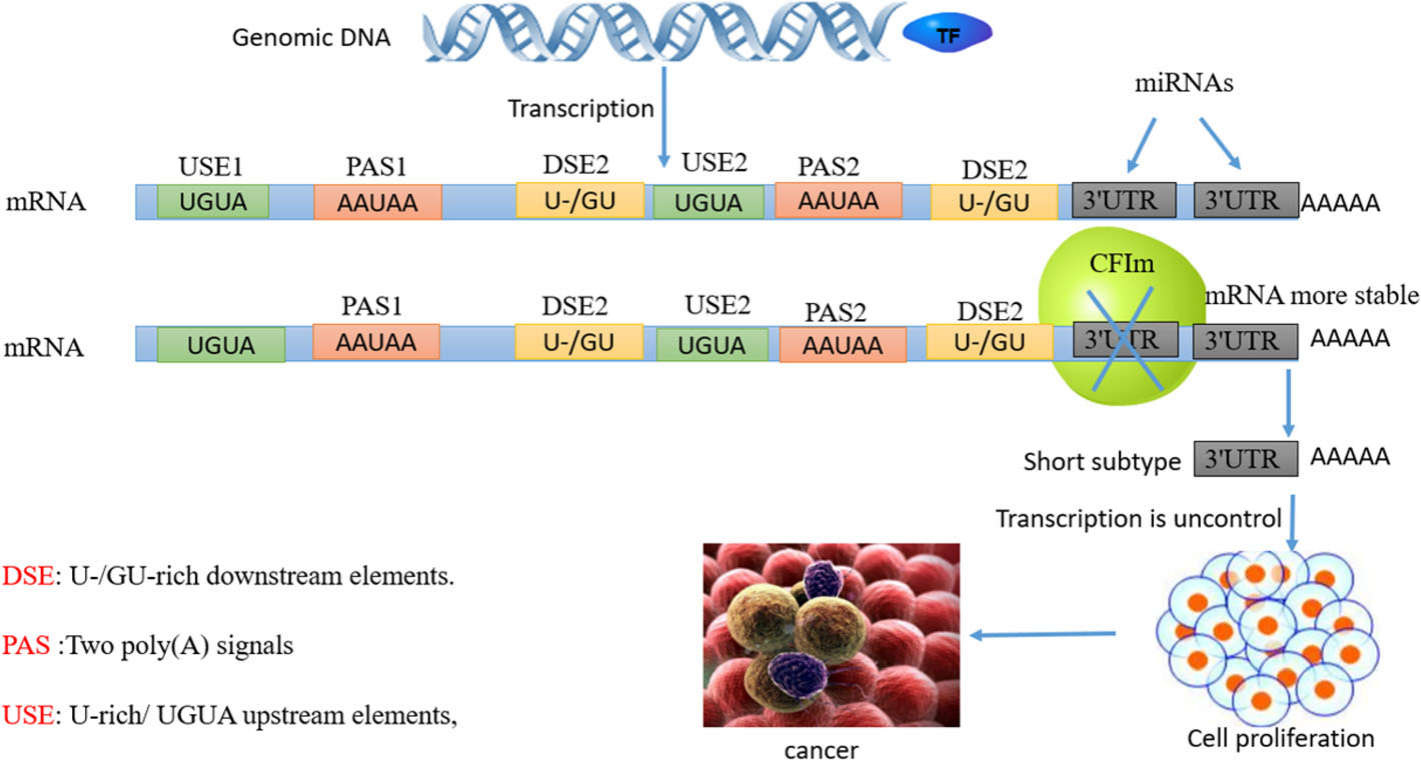
APA makes the miRNA binding site missing by a cleavage complex (CFIm), which makes the mRNA more stable, the translation efficiency is higher, and the transcription of the mRNA is out of control, thereby promoting the migration and invasion of tumor cells

Tumor cells express a large number of aberrant mRNAs. The length of these mRNA 3′UTRs is shorter than that in normal cells. Studies have revealed that this phenomenon is usually caused by APA [146–148]. Because 3′UTRs contain a large number of binding sites for negative regulators, the shorter 3′UTRs greatly improve the stability of mRNA and the protein expression by more than ten times [146, 149]. It has been indicated that the shortening of 3′UTRs is regulated by a cleavage complex (CFIm), and there are mainly three polypeptides CFIm25, CFIm59, and CFIm68 (Fig. 9) [150–152]. Recently, Masamha et al. [153] find that CFIm25 plays a carcinogenic role in gliomas, and knockdown of CFIm25 decreases the expression of terminal polyadenylation of cyclin D1 (CCND1) and increases the proliferation of tumor cells. In cancer, RBPs can also co-regulate mRNA with APA, such as cytosolic polyadenylation element-binding protein 1 (CPEB1), which regulates polyadenylation of transcripts, especially cell cycle-related mRNAs, including extending poly (A) tails and enhancing translation efficiency [154]. Studies have discovered that the binding of CPEB1 to mRNA can assist APA in its 3′UTR region [155]. Furthermore, CPEB1 recognizes the binding site of non-canonical poly (A) polymerase Gld2/4 and noradenylation enzyme PRN, and specifically binds to the cytoplasmic polyadenylation element (CPE), resulting in a shortened length of hundreds of mRNA 3′UTRs and promoting tumorigenesis [156]. For example, in hepatocellular carcinoma, CPEB1 can directly target the 3′UTR of SIRT1, control the poly (A) tail length, inhibit its translation, and negatively regulate the stemness and resistance of liver cancer [157]. In endometrial cancer, miR-183 inhibits CPEB1 expression at the transcription and translation levels, and targets CPEB1 to induce EMT and promote tumorigenesis of EC cells [158]. In addition, CPEB4, another member of the CPEB family, is highly expressed in melanoma, glioblastoma and pancreatic ductal adenocarcinoma [159, 160]. In melanoma cells, MITF and RAB72A are vital regulators of their progression. CPEB4 can control the polyadenylation of MITF and RAB72A, improve their translation efficiency and further enhance the cell proliferation capacity [160]. In pancreatic cancer, CPEB4 can prolong the poly (A) tails of TPA mRNA and activate its translation, thereby enhancing cancer cell growth, invasion and angiogenesis [159]. Recently, reports have shown that CPEB1 and CPEB4 co-regulate the post-transcriptional process of VEGF. First, activation of CPEB1 increases the APA of CPEB4 and VEGF expression and the dual activation of VEGF promotes tumor angiogenesis [161]. These studies indicate that CPEBs are likely to be potential therapeutic targets for angiogenesis-dependent diseases including cancer [154].

#### Stability

**Fig. 10:**
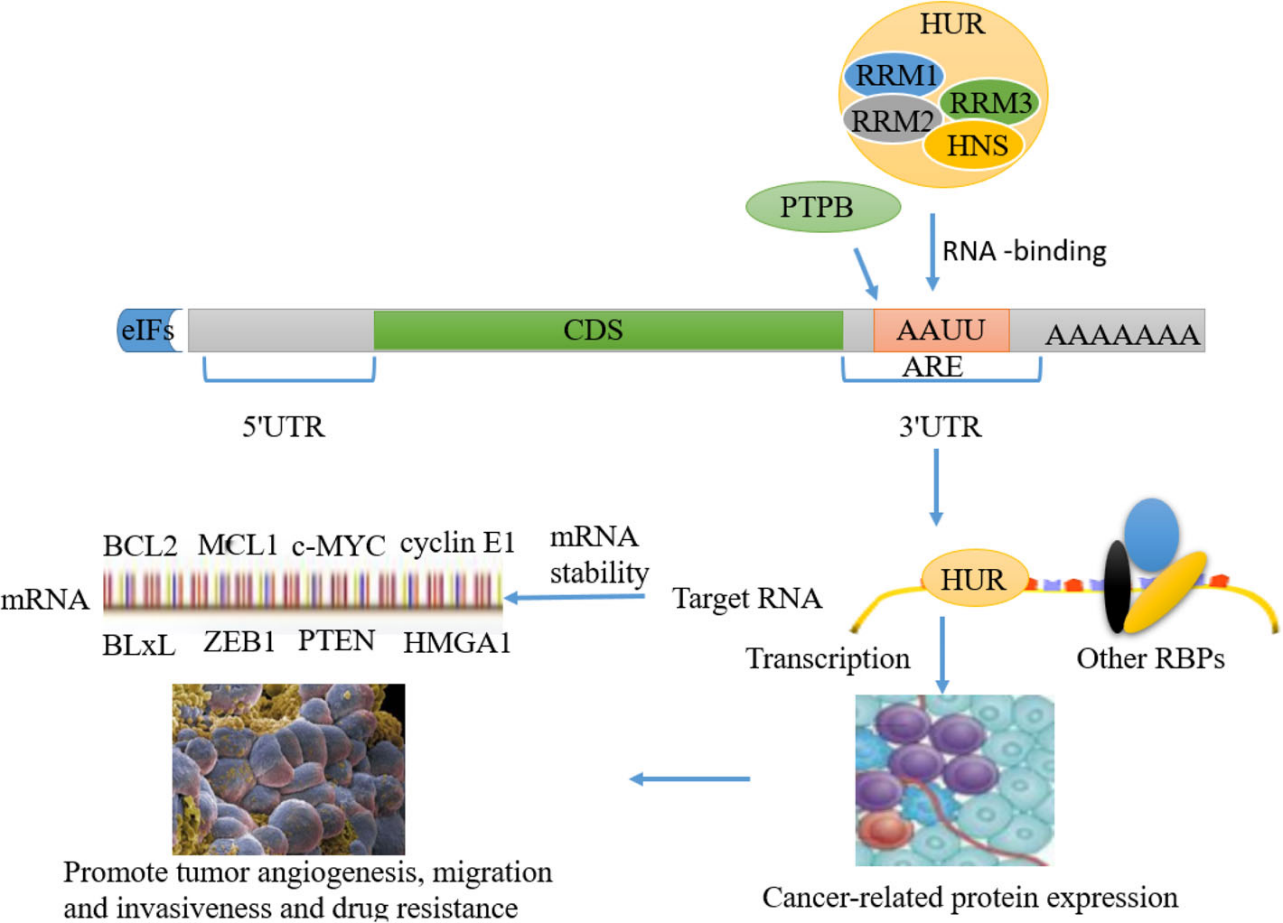
HUR, PTPB, or other RNA binding proteins enhance the stabilization of BCL2, MCL1, c-myc, cyclin E1, BLxL, and ZEB1 PTEN-related mRNAs by binding to the ARE sequence elements of mRNA in 3′UTR, thereby increasing the expression of cancer-related proteins and promoting tumor angiogenesis, migration, invasion and drug resistance

The transcription, translation, stability and subcellular localization of mRNA are closely related to the highly regulated RNA-binding protein (RBP), thereby exerting a variety of cellular functions. In addition to the poly (A) tail at the 3′ end, the structure that impacts the stability of the eukaryotic mRNA has a cap structure at the 5′ end. There are many ways to degrade mRNA, one of which is to start with the hydrolysis of poly (A) tail, and then process to 5′ end decapping and 5′ → 3′ direction degradation, or after poly (A) tail hydrolysis, degradation in the 3′ → 5′ direction [162]. To date, the structure involves in reverse degradation of mRNA is the AU-rich original (ARE) of 3′UTR, and it is estimated that 16% of the transcripts contain ARE [163]. When cells are not stimulated, ARE-containing mRNAs are degraded at any time by poly (A) tails. After cells are subjected to different stimuli, ARE-binding proteins (ARBPs) may promote mRNA degradation or improve their stability [164]. ARE is the most studied structure related to mRNA stability [165]. The most common RBPs that can stabilize mRNA are human antigen R (HuR), T cell intracellular antigen 1 (TIA-1), and TIA-1-related protein (TIAR) [166]. The abnormal increase and extension of ARE-encoded mRNAs are related to cancer (Fig. 10), including the ARE-containing transcripts, oncoproteins, growth factors, and their receptors and cyclins. Abnormal expression of mediators is closely related to angiogenesis, chemotaxis, and invasiveness, which suggests that mRNA stability plays an important role in tumorigenesis [167, 168].

On the one hand, RBPs can enhance the stability of mRNA to regulate the protein expression of its target genes. For example, the PTBP family belongs to RBPs, and there are three main members PTBP1, PTBP2, and PTBP3. PTBP1 and PTBP2 regulate mRNA splicing, stability, localization and translation [169]. However, PTBP3 can induce epithelial-mesenchymal transition (EMT) of breast tumor cells and promote their aggressive growth and metastasis. PTBP3 regulates the expression of the transcription factor ZEB1 by binding to the 3′UTR of its mRNA, thereby preventing its degradation [47]. Some studies have also indicated that HuR can regulate some apoptosis-related genes, such as stabilizing their mRNAs by binding to the 3′UTRs of MCL1, BCL2 and BLxL. This post-transcriptional regulation promotes the growth of glioma cells and heightens its resistance to multiple drug tolerability, including etoposide, topotecan, and cisplatin [170]. In addition, by binding to the 3′UTR of cyclin E1, which is overexpressed in breast cancer, HuR improves cyclin E1 mRNA stability and thus increases its protein expression [171]. In addition, the IGF2BPs family is important for tumor development through regulating mRNA stability. For example, IGF2BP1 can prevent the degradation of PTEN mRNA and promote the targeted migration of tumor cells [172]. IGF2BP1 can also stabilize c-MYC and MKI67 mRNA and promote their expression, thereby regulating the proliferation and apoptosis of liver cancer cells [173]. IGF2BP2 can stabilize the mRNA of oncogene HMGA1 and facilitate the proliferation of cancer cells [174]. At the same time, IGF2BP2 can suppress the degradation of RAF1 mRNA caused by miR-195 and enhance the viability of colorectal cancer cells [175]. On the other hand, RBPs can also accelerate the degradation of mRNA. For instance, MCPIP1 can selectively expedite the degradation of a series of anti-apoptotic mRNAs, including Bcl2L1, Bcl2A1, RelB, Birc3, and Bcl3, and induce apoptosis in breast cancer cells [176]. In the IGF2BPs family, IGF2BP1 can reduce the stability of HULC mRNA which is specifically and highly expressed in liver cancer [177]. IGF2BP3 can accelerate the degradation of EIF4E-BP2 mRNA and thus promote the proliferation of cervical cancer cells [178].

#### Subcellular localization

**Fig. 11:**
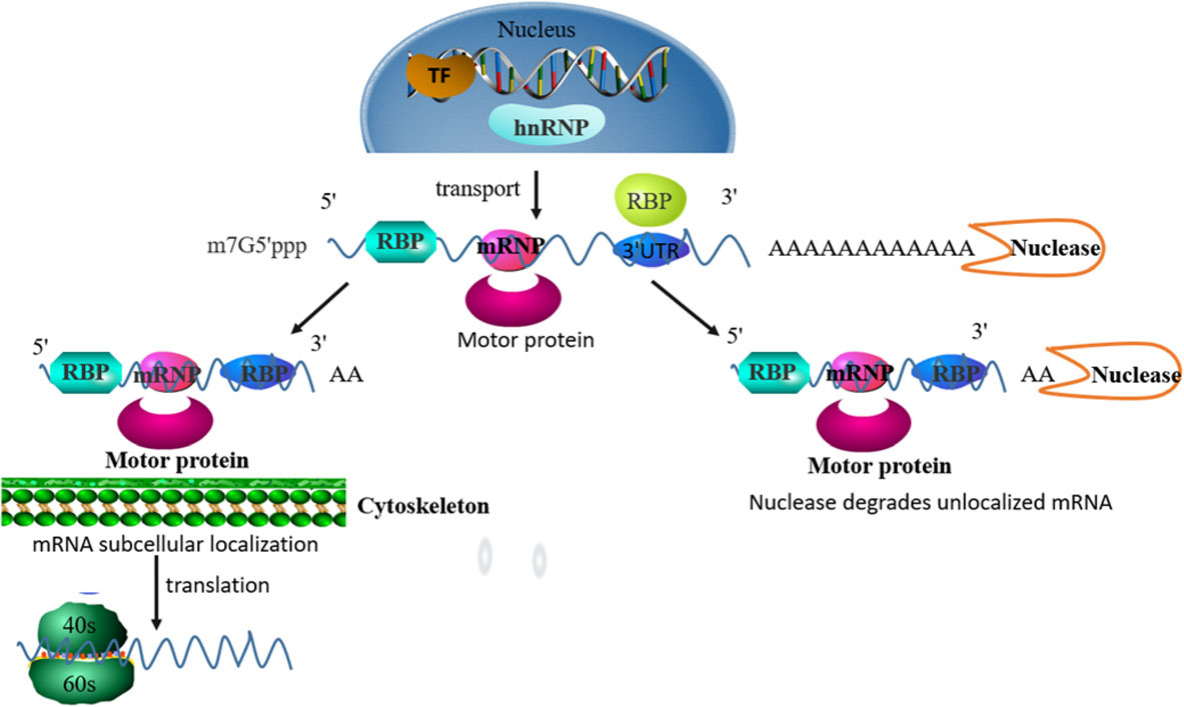
DNA is transcribed into RNA. With the help of hnRNA, pre-mRNA is transported to the cytoplasm. RBP binds to pre-mRNA and participates in splicing and splicing to form mRNA. Then hnRNA enters the cytoplasm and becomes mRNPs, which becomes a substrate for mRNA localization, and subsequently to be deadenylated so as to ensure that the mRNA can be effectively located to a specific position and efficiently synthesize protein

Some mRNAs are transcribed in the nucleus, then transported into the cytoplasm and translated to produce proteins. However, other mRNA transcripts directly target specific regions for local translation or distribution, resulting in an asymmetric distribution of cytoplasmic proteins [179]. The subcellular localization of RNA mainly recognizes the cis motif by RBP, or specific binding elements (zipcodes) in the 3′UTR of the target gene form a secondary structure, which serves as the binding site of RBP to regulate localization in the cell and then mediate the localization of RNA to a specific subcellular compartment [180]. First, hnRNPs in the nucleus recognize and bind to mRNA, and they are consigned into the cytoplasm from the nucleus. One part of the hnRNPs returns to the nucleus and the other forms a complex with the motor protein [179]. This mechanism is crucial for establishing and maintaining cell polarity, disruption of which can result in cancer progression [181] (Fig. 11). For example, The RBP Tia1 binds to a subset of cellular stress-related transcripts (p53 mRNA) and controls p53 mRNA translational silencing and RNA particle localization. When Tia1’s DNA is damaged, Tia1 dissociates from its mRNA target, p53 mRNA is released from the stress particles and binds to polysomes, resulting in the relocation and translation of mRNA [182]. TIA1 interacts with various tumor-related mRNAs and participates in cancer cell proliferation, apoptosis, invasion, metastasis, angiogenesis and immune escape [183, 184]. IGF2BP1, which is a member of the RBPs of the VICKZ family, is mainly composed of two RNA recognition motifs (RRMs) and four K homology (KH) domains. IGF2BP1 plays an important role in the process of carcinogenesis [185]. After transcription, IGF2BP1 can correct the mRNA expression of certain oncogenes, thereby promoting tumor cell proliferation and growth, invasion and chemical metabolism, which is related to a poor overall survival and metastasis of various cancers [186]. In polar cells, such as migrating fibroblasts and neurons, IGF2BP1 promotes the transport of ACTB transcripts to actin-rich processes [172]. In the beginning, IGF2BP1 in the nucleus is linked to the transcript of β-actin. After being transported to the cytoplasm, IGF2BP1 causes translational silencing of the mRNA but has no implication for its stability. Then mRNA is transported to the periphery of the cell; Src-directed tyrosine phosphorylation in the junction region of KF domains 2 and 3 of IGF2BP1 can induce the disassembly of cytoplasmic messenger ribonuclear protein and activate the translation of ACTB mRNA. Finally, the translation of mRNA starts and accelerates the accumulation of β-actin [187, 188]. In addition, IGF2BP1 is overexpressed in primary tumor tissues such as breast cancer, colon cancer and non-small cell lung cancer [189–191]. Downregulation of IGF2BP1 can affect the transport of adhesion and migration-related target genes, including α-actin, β-actin, E-cadherin and the Arp2/3 complex subunit Arp-16 [192, 193]. Therefore, the silencing of IGF2BP1 can destroy the connection between cells, decrease its adhesion ability, and promote cell migration and invasion. In addition, adenomatous polyposis protein (APC) is also an RBP whose function is related to cell localization. This protein is a positive microtubule end tracking factor and suppresses colon cancer progression. Recent studies have shown that APCs are not uniform as classical RBPs in their localization function. APC binds to 3′UTR of β2B-tubulin to regulate the location of cytoskeleton-related mRNAs. Studies have shown that the RNA binding region of APC contains cancer-related missense mutations and deletions [194].

#### Translation

**Fig. 12:**
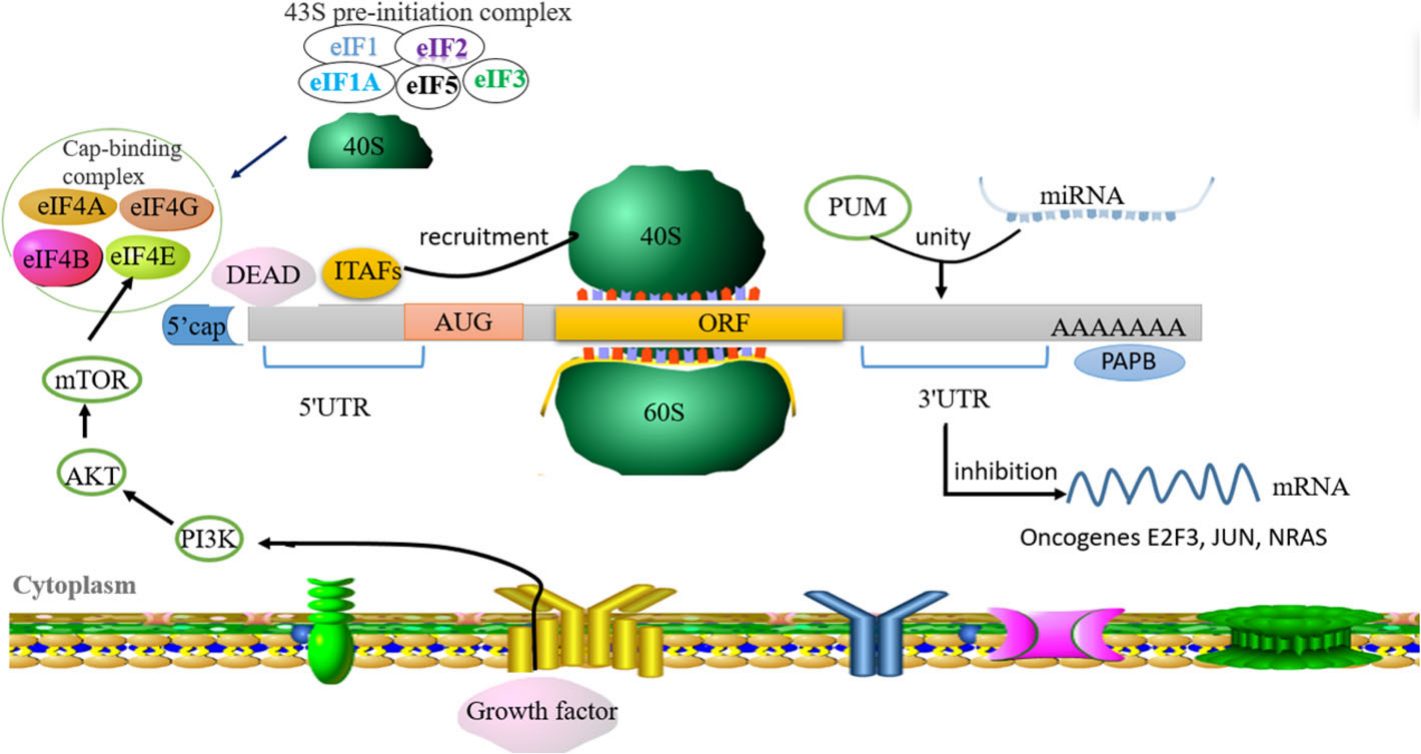
The mRNA translation process is mainly the 5′cap structure of the eIF4F cap-binding complex that recognizes and binds to the mRNA. When the 43S pre-initiation complex-mediated cap binding and ribosome binding, translation starts from the AUG. Abnormal mRNA translation of eIF4E, XIAP and LAMB1 is closely related to the occurrence of cancer. In addition, the binding of PUM and miRNAs to 3′UTR can inhibit the translation of E2F3, JUN, and NRAS mRNA and its overexpression is related to tumor development

The translation process is intricate and can consist of three stages: initiation, extension and termination. Translational regulation of most mRNAs occurs at the initial stage. RBPs, such as the 5′cap-binding complex eIF4F and poly (A)-binding protein (PABP), are required for cyclization and translational activation of mRNAs. In cancer, almost all key oncogenic signaling pathways are abnormal and cause translational disorders such as the PI3K/AKT/mTOR, RAS/MAPK and Wnt/β-catenin signaling pathways [195–197]. It has been reported that dysregulated expression of eIF4E, a component of the eIF4F complex, is associated with approximately 30% of human tumors [198]. In addition, it has been indicated that knockdown of eIF4E in mice not only sustain a normal physiological state but also manifest the significant inhibition of tumors [199]. These studies indicate that in mammalian cells, the expression of eIF4E is higher than normal for protein translation, and eIF4E can induce translation of oncogenic mRNAs and promote cancer development. During the gradual development of cancer, certain structural and sequence-specific regulatory elements can control protein translation [197]. As one of these organizational elements, the 5′UTR has received widespread attention. 5′UTR is an internal ribosome entry site (IRES) and can recruit ribosomes directly in combination with IRES transacting factors (ITAFs) to initiate translation in a hat-dependent manner. The occurrence of cancer is associated with dysregulation of hat structure-dependent translation. Research has shown that La ribonucleoprotein domain family member 3 (LARP3) can induce IRES-mediated translation of two genes, including anti-apoptotic XIAP and EMT-related LAMB1 mRNA, and enhance cancer cell survival and invasion [139–141] [200–202]. In addition to 5′UTR, 3′UTRs also play a major role in the regulation of the translation process. For example, DAB2 and ILEI genes encoding EMT-related proteins contain TGF-β-activated translation (BAT) stem-loop elements on their 3′UTRs [84, 85]. When the TGF-β signaling pathway is silenced, the BAT element recruits eukaryotic translation elongation factors 1α1 (eEF1A1) and hnRNPE1 to prevent binding to their 3-UTR BAT elements, which can mediate the translational silence of Dab2 and ILEI (two EMT transcripts). Upon the TGF-β signaling pathway is activated, hnRNPE1 is phosphorylated and sheds from the BAT element to promote the occurrence of EMT [85]. In addition, Rumis’ Pumilio (PUM) human ortholog family is also involved in the regulation of protein translation. It also enhances the activity of multiple E2F3 target microRNAs (miRNAs) by binding to E2F3 3′UTR. PUM inhibits the translation of proliferating cell transcription factor E2F3 protein; its overexpression is related to tumor development [203]. Studies have found that the 3′UTR regions of the oncogenes E2F3, JUN and NRAS on the regulatory axis are shortened, leading to the disappearance of the PUM binding site, which in turn triggers the expression of these three genes and promotes cell proliferation (Fig. 12) [204].

#### Chromosomal remodeling and DNA damage

RBPs are increasingly appreciated for their participation in the entire life course of RNA [205]. Recent studies have shown that in addition to participating in traditional RNA processing, RBPs locating at chromatin may also participate in chromatin remodeling and DNA damage repair in mammalian genomes [206, 207]. Chromatin immunoprecipitation sequencing (ChIP-seq) revealed the extensive interaction of RBP RBFox2 with chromatin, RBFox2 interacts with Polycomb complex 2 (PRC2) and H3K27me3 so as to maintain a key signal of gene-stable expression in the mammalian genome [208]. Another interesting phenomenon is that by comparing RBP and TF ChIP-seq signals in HepG2 cells, Xiao et al. found a co-existence by a large number of transcription factors, enhancers and RBP, indicating that they have a coordinated function at the chromatin level [209]. For example, transcription factor YY1 and RBP RBM25 have a coordinated function chromatin remodeling, as evident by the fact that knockout of RBM25 attenuates YY1-mediated effects on chromatin binding, DNA circularization, and transcription [209]. In addition, DNA double-strand damage repair is essential for maintaining genome stability and preventing developmental disorders, neurodegenerative diseases and cancer. There exists two main ways to repair DNA double-strand breaks: non-homologous end joining (NHEJ) and homologous recombination (HR). Increasing evidences show that RBPs are involved in DNA damage response (DDR) and DNA repair [210]. For example, RBMX is a hnRNP protein which regulates alternative splicing through RNA recognition (RRM) and promotes DNA damage repair through HR; this process is independent of ATM signaling and H2AX, but depends on PARP1 [211]; RBP HuR has been reported as a PAR-binding protein in response to DNA damage. HuR binds and stabilizes PARG mRNA, thereby increasing PARG expression and regulating PARP-1-chromatin dynamics, which contributes to DNA repair and the inhibition of PARP inhibitor resistance [212]. In addition, triple-negative breast cancer (TNBC) patients are usually treated with chemotherapy, but the repair of DNA double-strand breaks (DSB) in tumor cells may result in chemoresistance. Studies have shown that IGFBP-3 participates in the repair of DSB damage through NHEJ, responds to DNA-destructive chemotherapy, and forms a nuclear complex with EGFR and DNA-PKCs, promotes the stability of tumor cell genome and leads to the migration and invasion of tumor cells [213]. Moreover, poly (ADP-ribose) polymerase (PARP) interacts with RBPs and regulates various important cellular processes, such as coordinated regulation of single-stranded DNA repair and differentiation, cell proliferation, and tumor transformation [214]. Importantly, PARP1 inhibitors have been widely used for cases of BRCA1 mutation or dysfunction [215]. Notably, PARP inhibitors may inhibit DNA repair through the NHEJ pathway and cause increased DNA double-strand breaks, thereby inhibiting tumor cell proliferation and metastasis [216]. Clinical studies have shown that the PARP inhibitor veliparib (ABT-888) can enhance the effects of radiation therapy through promoting the accumulation of nuclear EGFR translocation and DNA damage [217]. Therefore, we believe that more studies should focus on the interaction of PARP and RBP, which jointly regulates tumorigenesis, and PARP inhibitors might be the critical partners for RBP-targeted therapy in the future.

## Conclusions

Notably, in recent years, reversible modification of N6-methyladenosine (m6A) on mRNA has been shown to be a common phenomenon [218]. Both mRNA and lncRNA have m6A modifications in large amounts. m6A can accelerate the processing time of mRNA precursors and the speed of mRNA transport and nucleation in cells [219]. Importantly, RBP is a key regulator of m6A’s biological function by regulating methyltransferase installation. The methyltransferase complex is mainly composed of methyltransferase-like 3 (Mettl3), Mettl14, and Wilms Tumor1-related proteins (WTAP) and subunits, including Virilizer, RNA-binding motif protein 15 and zinc finger protein Zc3h13 [220]. RBP-mediated m6A modification can also cause a series of diseases, including tumors, nervous system diseases and delayed embryonic development [221, 222]. For example, RBP IGF2BP recognizes the K homology (KH) domain through its m6A binding site and acts as an m6A reader. With the help of cofactors, IGF2BPs promote the stability of thousands of potential target mRNAs, hinder miRNA-induced RNA decay and improve the expression of oncogenes (MYC, Actin, Lin28, SRF) to play a carcinogenic role in cancer [223].

**Fig. 13:**
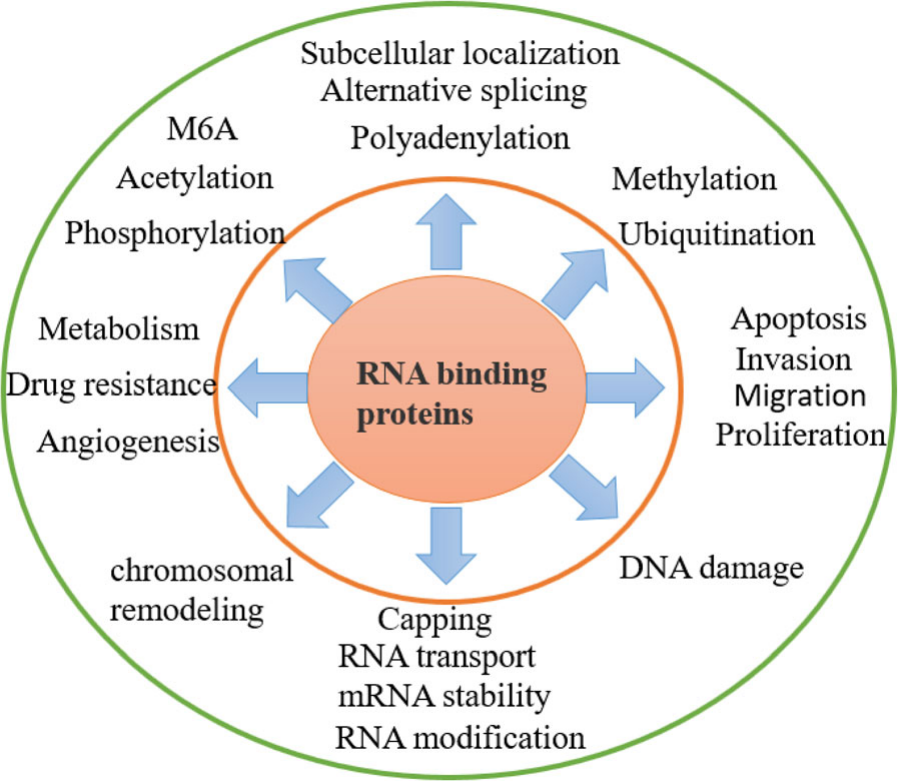
RBPs participate in the entire physiological process of RNA and play a key role in the function of RNA. In tumor cells, the abnormal function of RBPs makes tumor cells be highly heterogeneous

This article mainly discusses the structure and abnormal expression of RBPs in tumors and focuses on the molecular mechanism thereby they exert their effects in cancer. Since RBPs are involved in various processes of tumors, suggesting that RBPs participate in a very complex regulatory network and change numerous characteristics of tumors (Fig. 13). RBPs not only regulate the life course from DNA to RNA, but also participate in the energy metabolism program of cancer cells and evading immune surveillance by controlling the stability and/or translation of mRNA. Under carcinogenic conditions, RBP mainly affects cancer development by changing a large number of downstream target genes related to tumors, thereby expanding its impact on tumors through the “chain effect.” Therefore, the potential of RBPs in cancer biology will be unlimited. At present, small molecule inhibitors targeting RBPs are being explored (Table 2). Based on the above research results, more and more researchers have explored onco-RBPs-targeting cancer treatment [230]. In the future, the potential of RBPs as drug targets should be further tapped.

**Table 2:** Small molecule inhibitors targeting RBPs

**Inhibitor**	**Structure**	**Research status and clinical trials**	**Type of tumor**	**RBP**	**reference**
1632		pre-clinical	Hepatocellular carcinoma	LIN8	[224]
VPC-80051		pre-clinical	Castration-Resistant Prostate Cancer	hnRNPA1	[225]
L-norleucine		pre-clinical	breast cancer	hnRNPA2/B1	[226]
CMLD-2		pre-clinical	colon cancer	HuR	[227]
Resveratrol		Phase II	Melanoma	RBFox2	[94]
MS-444–		pre-clinical	Colorectal Cancer	HuR	[228]
C1632		pre-clinical	Multiple tumors	LIN8	[51]
BTYNB		pre-clinical	Melanoma and Ovarian Cancer	IMP1	[229]

As more and more cancer-related RBPs are discovered, identifying their target genes is needed. A key issue is that the binding of many RBPs to RNA does not depend on classical RBDs [231], so identifying RBP-RNA interactions is a challenging task. In establishing bioinformatics predictions, structural studies of ribonucleoprotein complexes can provide important data for new modeling algorithms created by bioinformatics tools and also point the way to identify the different roles of RBPs in normal and disease environments [232]. In addition, various evolving methodologies base on immunoprecipitation technology (including antibodies for immunoprecipitation) can be used for large-scale identification of RBPs genomes, their targets and binding sites [233]. Although this method is still in its infancy, it is likely to be widely used in a short time. Additionally, combined with the latest research progress, cell models are being used for RBP research, such as human tissue organoids, biomimetic microfluidic culture systems and tumor xenograft of patients. The molecular mechanisms by which RBPs and non-coding RNAs co-regulated a series of mRNAs are being deeply explored [234], which allows us to fully understand RBP-mediated regulatory network in tumors. Therefore, in order to reveal such a huge regulation, the integration of a large number of data resources and technologies is necessary. The advance of such related comprehensive analysis software will be a great challenge.

## Data Availability

Not applicable

## Abbreviations

RBPs: RNA-binding proteins
RRMs: RNA recognition elements
RBDs: RNA-binding domains
ncRNAs: Non-coding RNAs
lncRNA: Long non-coding RNA
hnRNPL: Heterogeneous nuclear ribonucleoprotein L
hnRNP: Heteronuclear ribonucleoprotein
PKM2: M2-type pyruvate kinase
HuR: Human antigen R
EMT: Epithelial-mesenchymal transition
ESRP1: Epithelial splicing regulatory protein 1
RBM38: RNA-binding motif protein 38
PD-L1: Programmed cell death-ligand 1
MSI1: Musashi1
PTM: Post-translational modifications
CBP: CREB-binding protein
PRMT1: Protein arginine methyltransferase 1
eIF4E: Eukaryotic translation initiation factor 4E
CNOT4: Ccr4-Not transcription complex subunit 4
DAB2: Tumor suppressor disabled-2
ILEI: Interleukin-like EMT inducer
PRMT1: Protein arginine methyltransferase 1
SF3B1: Splicing factor 3B subunit 1A
MDS: Myelodysplastic syndrome
SR: Serine/arginine
APA: polyadenylation
APC: Adenomatous polyposis protein
3′UTR: 3′untranslated regions
